# Prediction of drop size distribution and mean drop size in an L-shaped pulsed packed column using artificial neural network (ANN) model and semi-empirical correlation

**DOI:** 10.1038/s41598-025-10084-0

**Published:** 2025-07-17

**Authors:** Ali Ravandeh, Sajad Khooshechin

**Affiliations:** 1 Department of Chemical Engineering, Shiraz University, Shiraz, 71345 Iran; 2 Department of Chemical Engineering, University of Larestan, Lar, Iran

**Keywords:** Pulsed packed column, Artificial neural network, Drop size, L-shaped column, Software, Chemical engineering

## Abstract

This study proposes the use of artificial neural network (ANN) and semi-empirical models for predicting mean drop size and drop size distribution in an L-shaped pulsed packed extraction column under non-mass-transfer conditions, employing toluene-water (T/W) and n-butyl acetate-water (B/W) systems. The ANN model was trained using the Levenberg–Marquardt algorithm and demonstrated excellent predictive performance, achieving R^2^ values of 0.981 and 0.986 for drop size distribution and mean drop size, respectively. Furthermore, the ANN model demonstrated superior accuracy in predicting mean drop size, with an average absolute relative error (AARE) of only 2%, significantly outperforming the semi-empirical model’s AARE of 6%. Moreover, the ANN model significantly reduced the maximum prediction error in drop size distribution, particularly under conditions where the semi-empirical model showed poor performance. The findings underscore the critical role of pulsation intensity and interfacial tension in determining drop size. Notably, higher pulsation intensities were found to significantly reduce the influence of interfacial tension. Finally, new semi-empirical correlations derived from the experimental data were established to predict both mean drop size and drop size distribution.

## Introduction

Liquid-liquid extraction is an established separation method widely applied in hydrometallurgy, biotechnology, petrochemical industries, and nuclear engineering. Among various extraction equipment, pulsed columns have gained significant attention due to their enhanced mass transfer performance and operational flexibility^1–6^. These columns are generally categorized into vertical and horizontal types. While vertical columns are effective for large-scale operations, they face height limitations for indoor setups. Conversely, horizontal columns overcome space constraints but typically suffer from lower throughput^7,8^. To address these limitations, Akhgar et al.^9^ proposed a novel L-shaped pulsed column that combines the benefits of both orientations. In addition, the L-shaped pulsed column offers improved operational performance. Its mass transfer efficiency is not only comparable to that of vertical and horizontal designs but may be enhanced due to better phase contact and flow dynamics. Moreover, its energy consumption falls between that of vertical and horizontal columns, offering a balanced compromise between performance and operating cost^10^. It is important to highlight that in the studied configuration, the vertical section is positioned downstream of the horizontal segment. This arrangement promotes a natural draft within the horizontal section, enhancing the transport of dispersed phase droplets. Consequently, the holdup in the L-shaped column is higher than that observed in the horizontal column, leading to a delayed onset of flooding.

The L-shaped configuration exerts a notable influence on drop dynamics, particularly when packing is employed within the column. One of its main advantages is the generation of a more uniform drop size distribution across both the horizontal and vertical sections. This uniformity reduces the inconsistencies typically encountered in L-shaped pulsed sieve-plate columns, where differences in drop size between sections can lead to conflicting flow patterns and diminished extraction efficiency. In contrast, L-shaped packed columns exhibit significantly reduced drop size variation, resulting in more stable hydrodynamic behavior and enhanced mass transfer conditions. Moreover, the interplay between pulsation intensity and the packing structure promotes greater energy dissipation via the formation of smaller vortices. These vortices facilitate the breakup of larger droplets into smaller ones, thereby increasing drop dispersion and interfacial surface area for mass transfer^11^.

A critical design parameter in pulsed extraction columns is the drop size, which directly influences interfacial area, phase holdup, residence time, and ultimately mass transfer efficiency^12–18^. Table 1 summarizes key studies that have predicted drop size in different pulsed column configurations and system characteristics. As seen, most studies have focused on vertical or horizontal columns, with limited attention to L-shaped geometries.

**Table 1:** Overview of semi-empirical models for predicting drop size across various pulsed column configurations and system characteristics.

Study	Orientation	Column type	Drop size data	Dispersed phase	Solute	Effect of mass transfer
Spaay et al.^24^	Vertical	Packing	Experimental data	Toluene, butanol	–	✗
Kumar and Hartland^25^	Vertical	Sieve plate	Published experimental data	Kerosene, methyl isobutyl ketone, carbon tetrachloride, xylene, toluene, tributyl phosphate, n-butyl acetate	Acetone, iodine, nitrophenol	✓
Shreenivasulu et al.^26^	Vertical	Sieve plate	Experimental data	Kerosene	n-butyric acid	✓
Torab-Mostaedi et al.^1^	Vertical	Packing	Experimental data	Toluene, n-butyl acetate	Acetone	✓
Samani et al.^27,28^	Vertical	Packing	Experimental data	Kerosene, toluene, n-butyl acetate	–	✗
Khajenoori et al.^7^	Horizontal	Sieve plate	Experimental data	Kerosene, toluene, n-butyl acetate, butanol	–	✗
Panahinia et al.^29^	Horizontal	Sieve plate	Experimental data	Toluene, n-butyl acetate, butanol	Acetone	✓
Amani & Esmaieli^30^	L-shaped	Sieve plate	Experimental data	Toluene	Acetone	✓
Khooshechin et al.^19^	L-shaped	Packing	Experimental data	Toluene, n-butyl acetate	Acetone	✓

Accurate prediction of drop size and its distribution is therefore essential for reliable column design and operation. However, due to the nonlinear nature of droplet formation and breakup, traditional semi-empirical models often fall short in delivering precise predictions across varied operating conditions. This challenge is particularly evident in L-shaped pulsed packed columns, where flow behavior is further complicated by geometry^19^.

With the increasing advancement of artificial intelligence technologies, researchers have widely employed machine learning techniques to address complex nonlinear problems in chemical engineering, particularly in modeling multiphase flow systems. Among these, artificial neural networks (ANNs) have gained considerable attention due to their ability to capture intricate, multidimensional relationships between operational parameters and system responses. Applications of ANN have been reported in predicting hydrodynamic parameters in various types of extraction columns. Specifically, ANN models have been applied to estimate drop size in pulsed disc and doughnut columns (PDDC) and pulsed sieve plate columns, often demonstrating superior prediction accuracy over conventional models^2,17,20^.

Hemmati et al.^2^ developed ANN model to predict holdup, slip velocity, and characteristic velocity in PDDC. Two different network architectures were evaluated: one consisting of two hidden layers with 35 and 5 neurons, and another with a single hidden layer containing 85 neurons. The results demonstrated that the dual-layer architecture provided more accurate and reliable predictions compared to the single-layer configuration.

Brockkötter et al.^21^ employed the ANN model to overcome the limited accuracy of semi-empirical correlations in predicting flooding behavior in pulsed sieve plate extraction columns. The ANN architecture comprised two hidden layers, each containing 75 neurons, and a root mean square error on the test dataset, indicating good predictive performance.

Wang et al.^20^ introduced a droplet breakage frequency model based on ANN in the PDDC, trained using experimental data obtained in their earlier investigations. The selected network architecture comprised two hidden layers, each containing seven neurons. Their findings demonstrated that the ANN model could accurately predict droplet size distributions across a range of operating conditions, showing strong agreement with the experimental observations.

Jin et al.^22^ developed a predictive ANN model for dispersed phase holdup in PDDC, utilizing an extensive experimental dataset compiled from multiple independent studies. Their systematic evaluation of network architectures demonstrated that a configuration employing 33 neurons in the hidden layer minimized the mean relative error (MRE) across all test cases.

Wang et al.^17^ used the ANN model to predict droplet size in PDDC and pulsed sieve-plate column. For PDDC, they applied a two-hidden layer architecture with 18 and 9 neurons, while a single hidden layer with 7 neurons was used for the sieve plate column. The ANN model demonstrated prediction accuracy with 9.6% average absolute relative error (AARE) for drop size estimation in PDDC, while showing 18.5% AARE in pulsed sieve plate column.

Although numerous semi-empirical and ANN-based models have been developed for conventional pulsed extraction columns, predicting drop size in L-shaped pulsed packed columns remains a major challenge. This is primarily due to the unique flow behavior and geometric complexity introduced by the L-shaped configuration, which causes the operating parameters to influence drop size differently in the vertical and horizontal sections. Consequently, correlations derived for either vertical or horizontal columns cannot be reliably extended to L-shaped systems. Furthermore, to date, no specific ANN model has been proposed for predicting drop size in L-shaped pulsed packed columns, and only one semi-empirical correlation exists under conditions involving mass transfer. While ANNs have been used for predicting drop size in pulsed extraction columns, their application to L-shaped configurations remains limited. This limitation arises because the optimal ANN architecture is highly dependent on the specific geometry of the column and the nature of the input and output variables, making it necessary to design and train separate models for each column type. Moreover, as highlighted in the review by Yadav and Patwardhan^23^, the validity of existing models is generally confined to the specific column designs and chemical systems for which they were developed, thereby restricting their applicability to other configurations. To address this gap, the present study:

- Developed novel semi-empirical correlations and ANN architectures based on experimental data from toluene-water and n-butyl acetate-water systems to accurately predict the mean drop size and drop size distribution even on conditions where conventional methods were previously weak in predicting it.
- Quantitatively evaluated the sensitivity of drop size to operational parameters and physical properties.
- Compared the predictive performance of the proposed models using various statistical metrics.

## Experimental

### Fluid systems

This study utilized toluene-water (T/W) and n-butyl acetate-water (B/W) systems, as suggested by the European Federation of Chemical Engineering for liquid–liquid extraction research^31^. Among various physical properties, interfacial tension has been identified as the most influential factor affecting droplet size, while the effects of density and viscosity are relatively minor^19^. Accordingly, the toluene-water and n-butyl acetate-water systems were selected due to their wide range of interfacial tension values (13.5–35.4 mN/m), allowing for a more accurate assessment of its impact on droplet behavior in the L-shaped column. In both systems, distilled water was the continuous phase, while the organic compounds toluene and n-butyl acetate, each with a purity of 99.5% by weight and purchased from Merck Company, were used as the dispersed phase. Interfacial tension was measured using a Krüss tensiometer, while the densities and viscosities of the organic and aqueous phases were determined using a pycnometer and a DVI-Prime viscometer, respectively. All experiments were carried out at a temperature of 20 ± 1 °C, and the physical properties of these systems at this temperature are detailed in Table 2.

**Table 2:** Physical properties of proposed systems at 20 $^\circ$ C ^31^.

Physical property	T/W	B/W
${\rho }_{c} (kg/{m}^{3})$	998	997.6
${\rho }_{d} (kg/{m}^{3})$	864	880
${\mu }_{c}$ $(mPa.s)$	0.963	1.027
${\mu }_{d}(mPa.s)$	0.586	0.734
$\sigma (mN/m)$	35.4	13.5

### Description of equipment

**Fig. 1:**
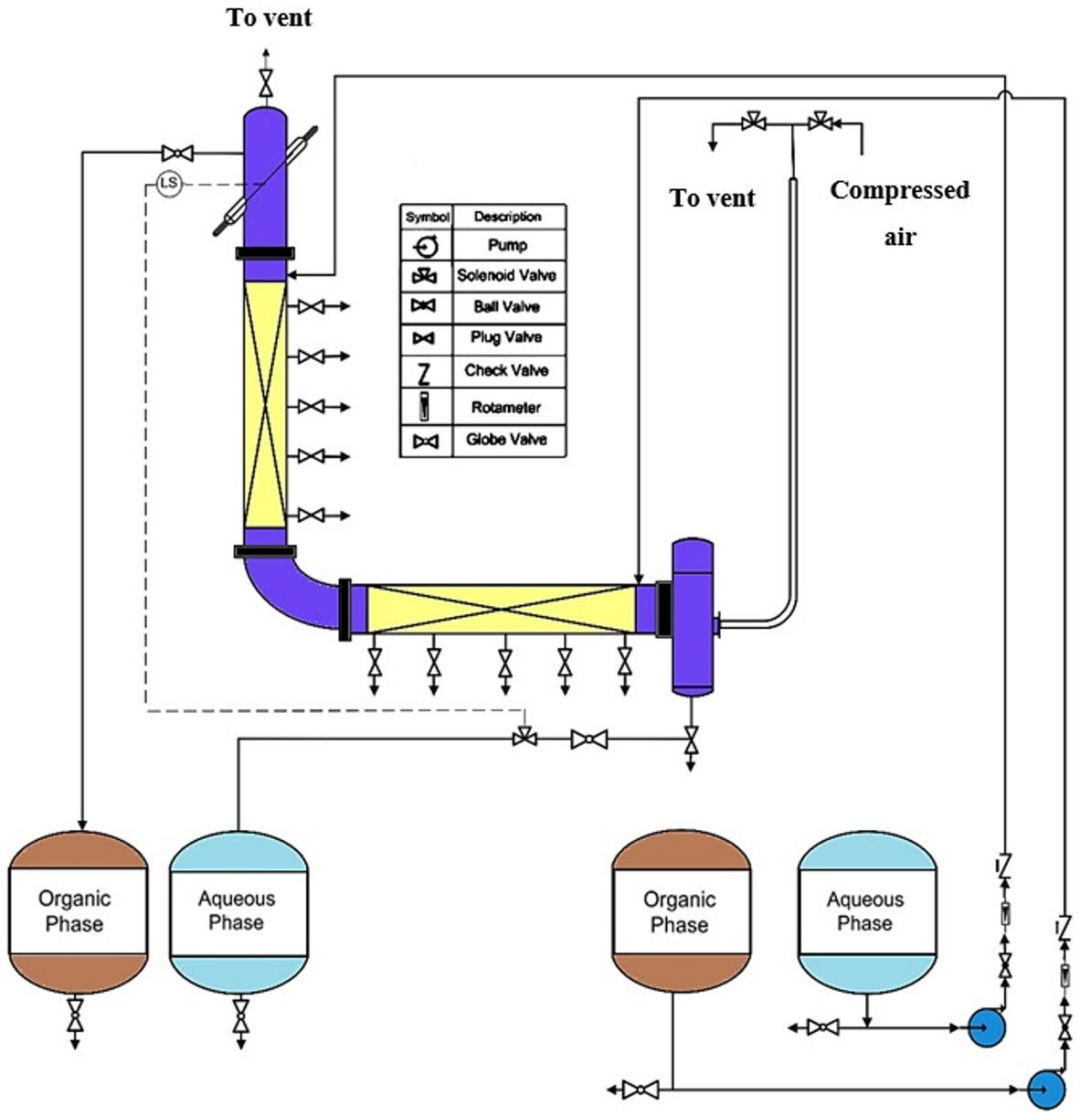
A diagram of the L-shaped pulsed packed column.

The L-shaped pulsed packed extraction column utilized in this research is shown in Fig. 1. The apparatus included vertical and horizontal sections filled with packing material to a height of 1.3 m, along with upper and lower settlers, four tanks, two pumps, two rotameters, and an air pulsation system. The operational segment of the column featured an internal diameter of 0.06 m and was constructed from glass. Ceramic Raschig rings, characterized by a porosity of 60% and a diameter of 0.63 cm, served as the packing material. To mitigate channeling of the dispersed phase flow, six baffles were installed in the horizontal section. The column’s inlets and outlets connected to four tanks. An optical sensor managed the position of the interface between the two phases at the column’s top. When adjustments to the interface position were necessary, an electronic signal triggered a solenoid valve to control the outlet flow rate. Additionally, the organic phase exited the column through an overflow. The pulsation intensity, set to the required amplitude and frequency, was controlled by compressed air that was repeatedly pushed into the air leg connected to the lower settling area of the column.

### Procedure

Initially, both phases were fully saturated with each other. Subsequently, the aqueous phase filled the column, and the pulsation intensity was adjusted to the desired amplitude and frequency. The organic phase was then introduced into the column. After setting the flow rates of each phase to meet the operational conditions, the system was allowed to stabilize for approximately 120 mins. The experimental ranges of the input operating parameters examined in this study are listed in Table 3. To evaluate the size of droplets in the dispersed phase, a photographic technique was employed using a Nikon D5000 digital camera. Six equidistant zones (three vertical and three horizontal) within the active section of the column were selected for image capture. The resulting photographs were analyzed using AutoCAD software to quantify droplet sizes. To ensure measurement accuracy, calibration was performed based on visible reference lengths in the captured images, such as the known column diameter and packing size. These references were used to scale the images in AutoCAD and to correct for any optical distortion caused by lens effects or perspective errors. This ensured consistency and reliability in the measured droplet sizes under experimental conditions. Approximately 1500 droplets were analyzed per experimental run to ensure statistical significance. This sample size was determined through preliminary assessments to provide a reliable estimation of the drop size distribution while minimizing the influence of random measurement errors. The large dataset enables a robust representation of droplet characteristics. While most of the observed droplets exhibited a predominantly spherical shape, ellipsoidal droplets were also identified in some cases. These were characterized by their major axis (R_2_) and minor axis (R_1_) as follows^30^:

1 $$d_{i} = \, \sqrt[3]{{(R_{{\text{2,i}}}^{2} \times R_{{\text{1,i}}} )}}$$

**Table 3:** Experimental ranges of the input operating parameters.

Liquid-liquid system	$Af \times 10^{2} (m/s)$	$Q_{d} (lit/h)$	$Q_{d} (lit/h)$
Toluene-water	1.2–1.6	1–4	1–4
n-butyl acetate-water	1–1.4	1–4	1–4

Investigations regarding the influence of column geometry on the pulsed extraction process indicated that the diameter of the column had a negligible effect on droplet size. This was attributed to the fact that pulsation intensity significantly influenced droplet size within the pulsed column. The energy input in this configuration was uniformly distributed across the column’s cross-section, which implied that droplet sizes near the wall were not expected to differ substantially from those in the center^23,32^.

After the drop sizes were measured, the Sauter mean drop diameter (d_32_) was employed to assess the mean drop size under the experimental conditions, as described by the following equation:

2 $$d_{32} = \frac{{\mathop \sum \nolimits_{i = 1}^{n} n_{i} d_{i}^{3} }}{{\mathop \sum \nolimits_{i = 1}^{n} n_{i} d_{i}^{2} }}$$

where *n* represents the number of droplets, and *d* is the drop diameter.

In this study, 18 experiments were conducted for both systems (toluene and n-butyl acetate), totaling 36 tests, to assess the influence of operational parameters (pulsation intensity, continuous and dispersed phase flow rates), and physical properties on drop size behavior. The central composite design (CCD) method was employed to systematically vary the operational parameters in 15 different conditions, with one test repeated four times at the center point to ensure system accuracy. The error in the system was determined to be approximately 5.0 %.

## Methodology

### ANN model

ANN is a computational approach used to model complex systems, particularly nonlinear systems, by learning from training datasets. ANNs use interconnected neurons to interpret and adapt to data patterns^33,34^.

**Fig. 2:**
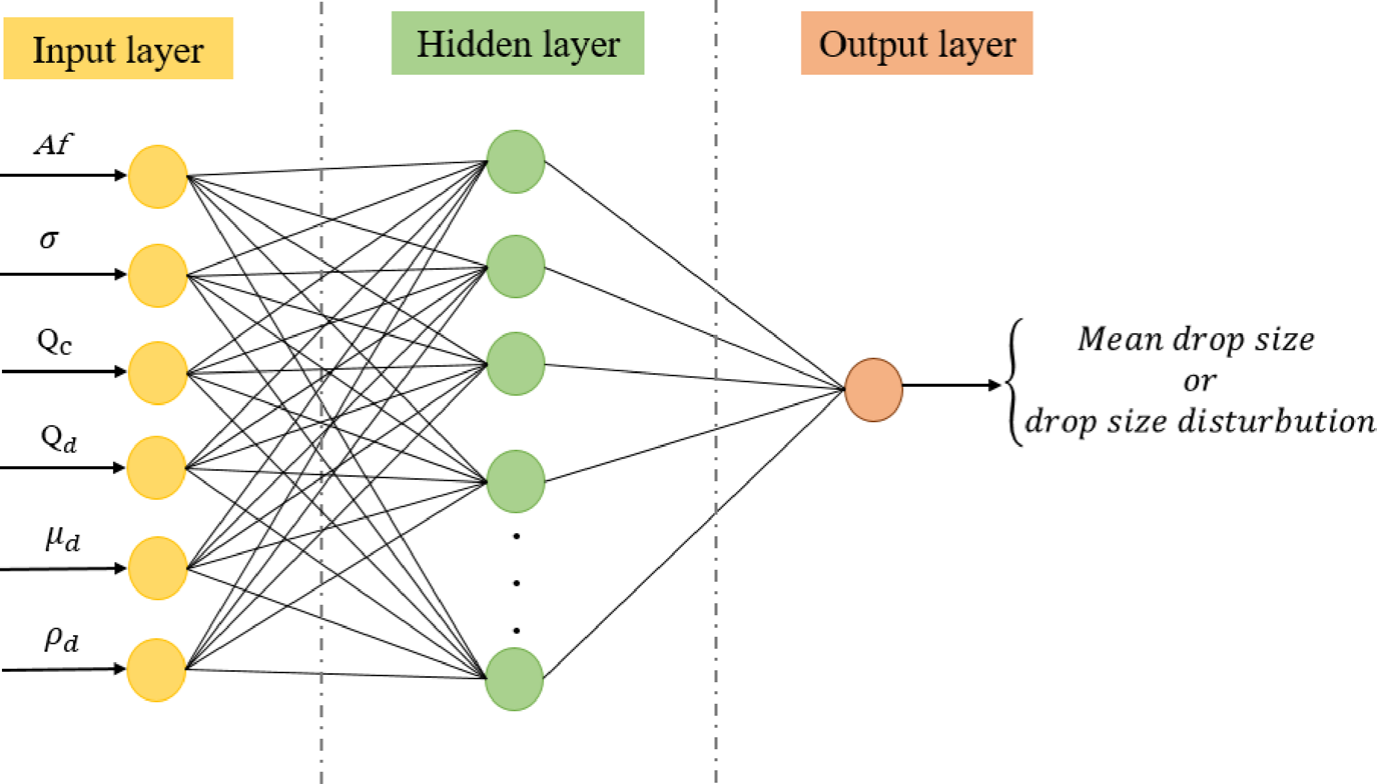
Schematic diagram of the ANN architecture.

ANN consists of input, one or more hidden layers, and an output layer as shown in Fig. 2^35^. In this study, the input parameters consisted of pulsation intensity, interfacial tension, flow rates of both continuous and dispersed phases, viscosity of dispersed phase, and dispersed phase density. Since the densities and viscosities of the continuous phase were nearly the same in both systems, their influence on the model output was disregarded. The mean drop size and drop size distribution were analyzed, with each considered as an output. To connect inputs to outputs, neurons in each layer play a key role by carrying out a series of operations. Initially, it sums the inputs after applying weights to them, according to the following equation:

3 $$net=\left(\sum_{i=1}^{n}{w}_{i}{x}_{i}\right)+b$$

where $net$ represents the neuron’s output, ${x}_{i}$ input, ${w}_{i}$ denotes the weighted coefficient for the neuron’s i^th^ input, and $b$ is the bios value.

The output values ($y$) are then processed through a transfer function ($f$) using the Eq. (4).

4 $$y=f(net)$$

Several transfer functions, including linear, hyperbolic tangent sigmoid, and logarithmic sigmoid, are summarized in Table 4 .

**Table 4:** Common transferr functions in ANN model.

Transfer function	Formula
Linear (purelin)	$f(x)=x$
Hyperbolic tangent sigmoid (tansig)	$f\left(x\right)=\frac{2}{{e}^{-2x}+1}-1$
Logarithmic sigmoid (logsig)	$f\left(x\right)=\frac{1}{{e}^{-x}+1}$

### Performance and training of the ANN model

Studies have suggested that an ideal evaluation of model performance should include at least one "goodness-of-fit" or relative error metric, such as the coefficient of determination (R^2^), and at least one absolute error metric, such as the mean square error (MSE) or the mean absolute error (MAE)^36^. In this study, the performance of the developed ANN model was assessed using three statistical criteria: MSE, MAE, and R^2^. These criteria are evaluated using the following equations:

5 $$MSE = \frac{1}{n}\sum\limits_{i = 1}^{n} {\left( {y_{\exp ,i} - y_{prd,i} } \right)}^{2}$$

6 $$MAE = \frac{1}{n}\sum\limits_{i = 1}^{n} {\left| {y_{\exp ,i} - y_{prd,i} } \right|}$$

7 $$R^{2} = 1 - \frac{{\sum\limits_{i = 1}^{n} {(y_{prd,i} - y_{\exp ,i} )^{2} } }}{{\sum\limits_{i = 1}^{n} {(y_{prd,i} - y_{m} )^{2} } }}$$

where ${y}_{prd,i}$ represents the calculated data from the model, ${y}_{exp,i}$ stands for the experimental data, ${y}_{m}$ is the average of the experimental data, $n$ and denotes the amount of data.

The most common training method in ANNs, the backpropagation (BP) algorithm, was employed for this study with a two-layer network using MATLAB^®^ Toolbox V9.1. The ANN model was created based on data derived from 36 experimental sets that included toluene-water and n-butyl acetate-water mixtures. Data normalization is important when variables are measured in different units; therefore, all the data was scaled within the range of 0–1. The data was divided into three distinct sets: training, testing, and validation, with the share of each set for both mean drop size and drop size distribution analyses presented in Table 5.

**Table 5:** Percentages of data used for training, testing, and validation.

Analysis type	Percentage of samples used in each set		
	Training	Testing	Validation
Mean drop size	70	15	15
Drop size distribution	80	10	10

## Results and discussion

### Selection of transfer function

The comparison of transfer functions was performed for both the mean and distribution of drop sizes, as shown in Table 6. According to the table, the “tansig” function yielded the lowest MAE and the highest R^2^ compared to the other functions, thereby justifying its use in the hidden layers. For the output layer, where values are expected to fall between 0 and a positive real number, the “purelin” function was chosen^37^. This choice was made because the “tansig” and “logsig” transfer functions generate values within limited ranges, making them unsuitable for the output layer.

**Table 6:** Comparison of different transfer functions.

Transfer function	Mean drop size		Drop size distribution	
	MAE	R^2^	MAE	R^2^
Linear (purelin)	0.101	0.907	0.015	0.918
Hyperbolic Tangent Sigmoid (tansig)	0.042	0.986	0.011	0.981
Logarithmic Sigmoid (logsig)	0.196	0.886	0.012	0.947

### Selecting backpropagation (BP) algorithm

Several common backpropagation (BP) algorithms were compared to identify the best fit for the data in Table 7. This table demonstrates that the Levenberg–Marquardt algorithm achieved the lowest MAE values of 0.016 and 0.011, as well as the highest R^2^ values of 0.986 and 0.981 for the mean drop size and drop size distribution, respectively.

**Table 7:** Comparison of different BP algorithms.

BP algorithm	Mean drop size		Drop size distribution	
	MAE	R^2^	MAE	R^2^
Levenberg–Marquardt	0.016	0.986	0.011	0.981
Powell–Beale conjugate gradient	0.045	0.933	0.012	0.974
One step secant	0.061	0.890	0.019	0.799
Polak–Ribiere conjugate gradient	0.060	0.881	0.012	0.976
Scaled conjugate gradient	0.060	0.864	0.012	0.977
Fletcher–Reeves conjugate gradient	0.082	0.795	0.012	0.977
BFGS quasi-Newton	0.094	0.736	0.018	0.840
Resilient (Rprop)	0.094	0.699	0.013	0.961

### Optimization of the ANN neurons

**Fig. 3:**
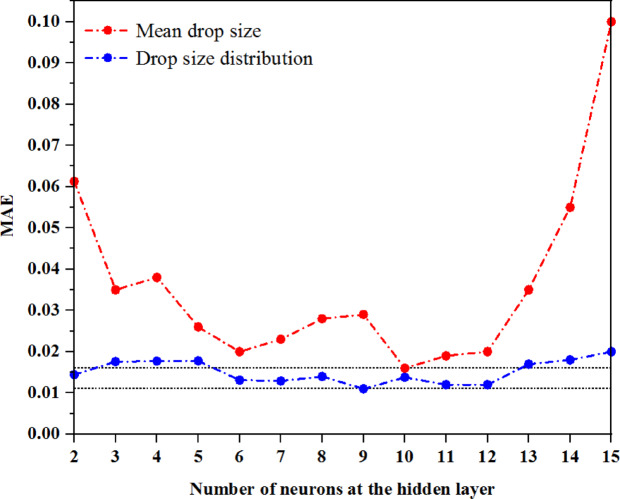
Dependence of MAE on the number of neurons.

The optimal number of neurons for the ANN model was identified by minimizing the MAE across the training, validation, and testing sets. The optimization process began by initializing the hidden layer with two neurons. As the number of neurons increased, the network reached several local minima, leading to fluctuations in the overall MAE. Figure 3 shows the relationship between the number of neurons and the MAE using the Levenberg–Marquardt algorithm, which was selected as the best BP method. As shown in this figure, the lowest MAE occurred at neuron numbers 9 and 10 in the hidden layer for the drop size distribution and mean drop size, respectively.

**Fig. 4:**
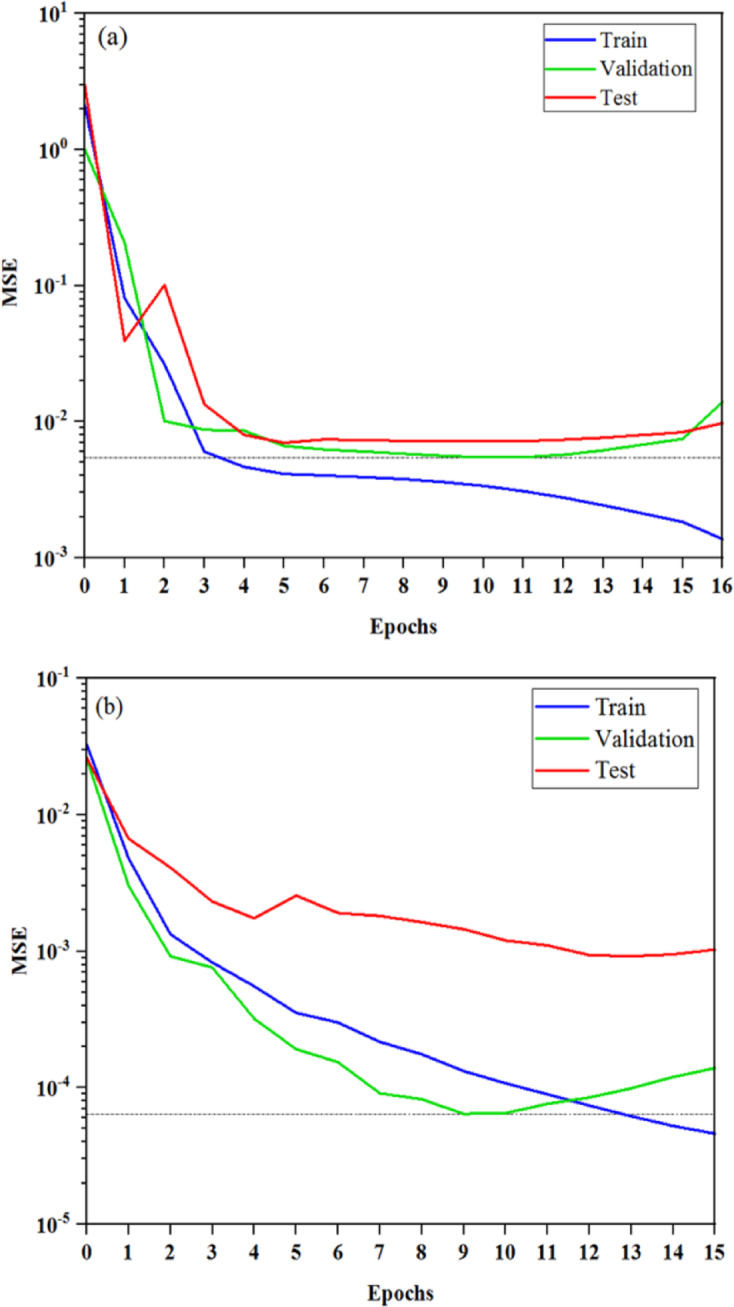
MSE for training, validation, and testing using the best-performing algorithm: (**a**) Mean drop size, (**b**) Drop size distribution.

Figure 4 illustrates the MSE during training, validation, and testing using the best-performing algorithm. As shown in this figure, the optimal MSE of validation was achieved at 10 epochs for the mean drop size and 9 epochs for the drop size distribution. Early stopping was applied to prevent overfitting, as evidenced by the increasing gap between training and validation errors.

### Regression analysis

**Fig. 5:**
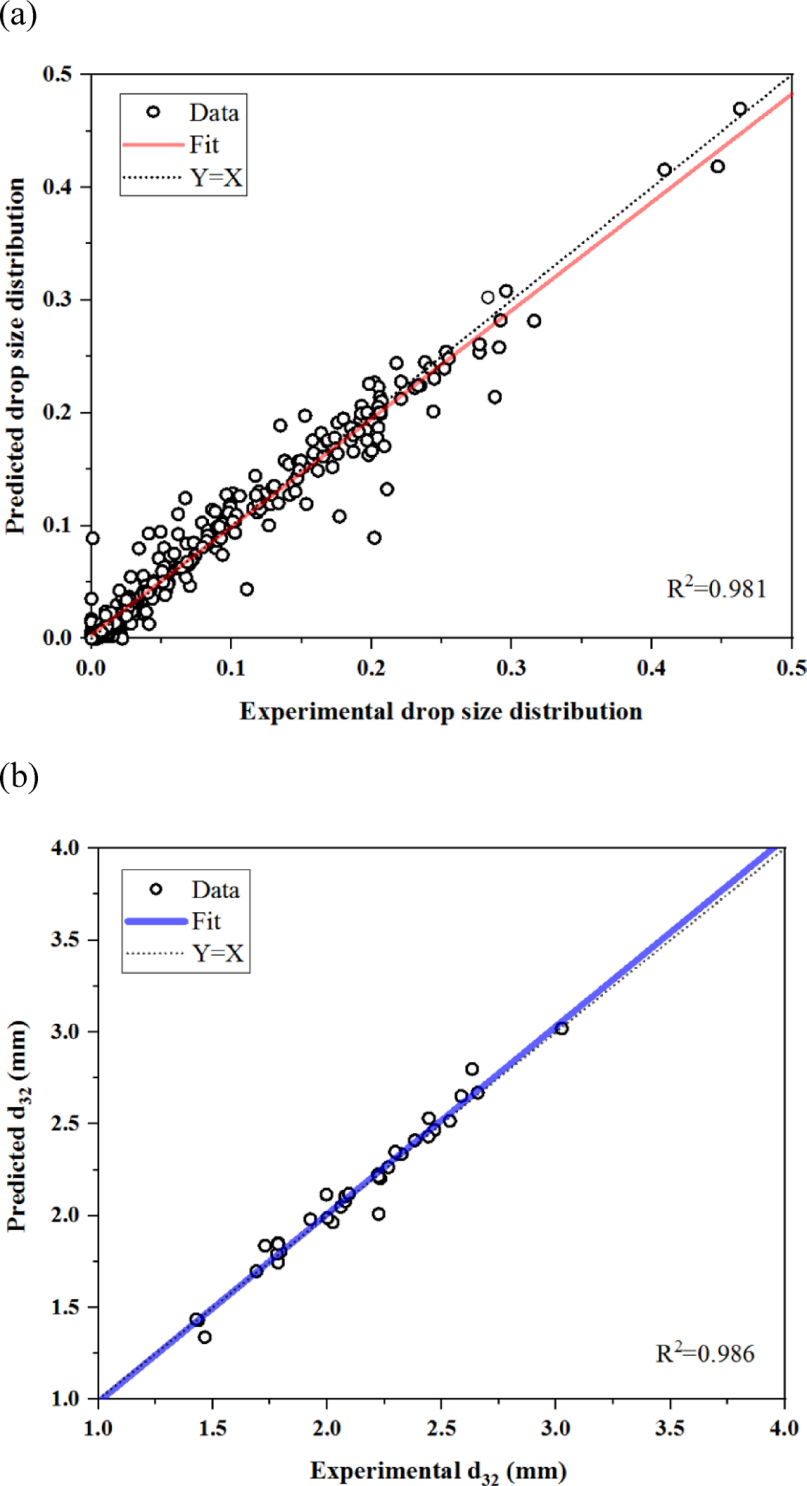
Comparison of ANN outputs with the measured data for: (**a**) drop size distribution, (**b**) mean drop size.

A regression analysis between the ANN outputs and the measured data was performed, as illustrated in Fig. 5. Despite the non-linear nature of the data, linear regression demonstrates a strong correlation between the ANN predictions and the measured values. The best linear fit, represented by a solid line, yields R^2^ values of 0.986 and 0.981 for the mean and distribution of drop size, respectively.

### The effect of operation conditions and physical property on drop size distribution

**Fig. 6:**
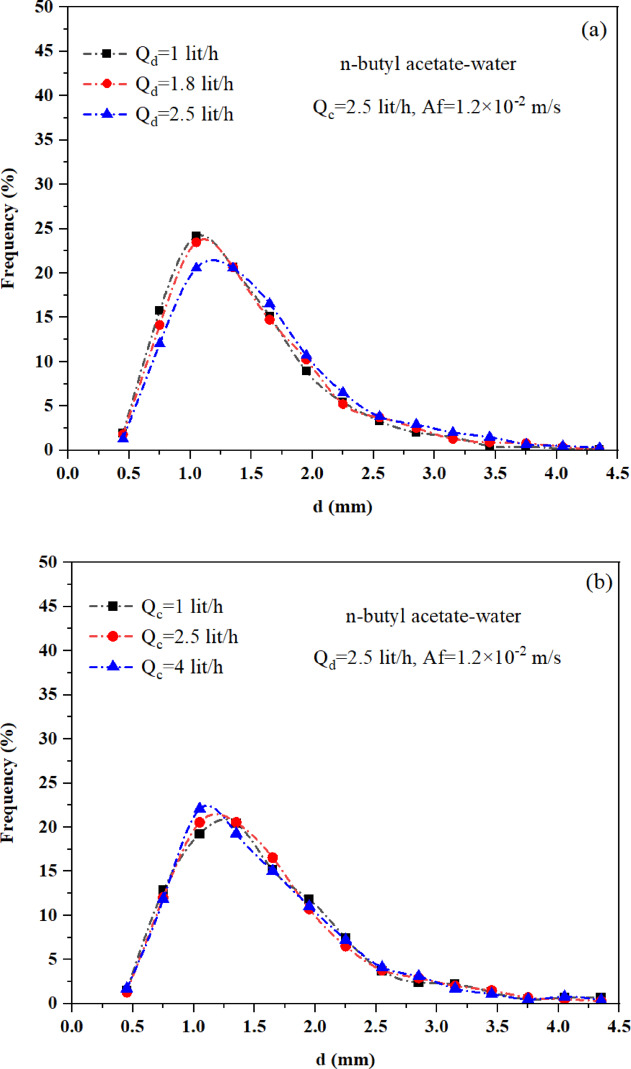

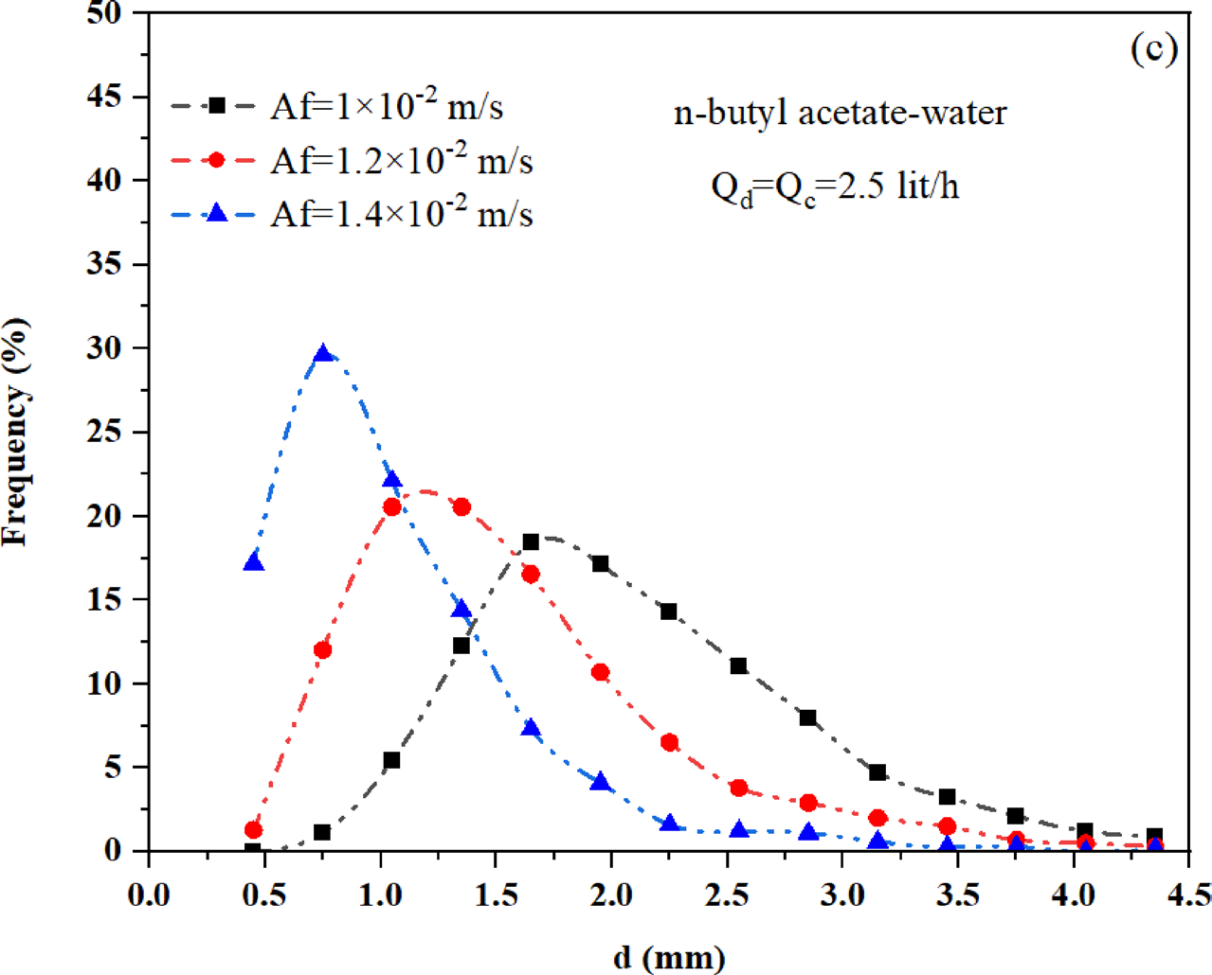
Impact of operational parameters on the drop size distribution for n-butyl acetate-water: (**a**) influence of dispersed phase flow rate, (**b**) influence of continuous phase flow rate, and (**c**) influence of pulsation intensity.

The column contains droplets of varying sizes. The drop size distribution is generally influenced by the balance between coalescence and breakage. However, experimental observations show that drop breakage plays a dominant role in determining the drop size distribution in a pulsed packed column. During the pulsations, droplets are dispersed, while the packing material inhibits their coalescence. Consequently, the drop size distribution in the pulsed packed column is expected to be narrower than in other types of pulsed columns, with a shift toward smaller drop sizes along the column. This study investigates how operational parameters, and physical properties affect the drop size distribution. The results of this study confirm that the drop size distribution is identical in both the vertical and horizontal sections of the L-shaped packed column. As demonstrated by Khooshechin^19^, this uniform distribution in both sections results from the use of packing within this column. Figure 6 illustrates the impact of operational parameters, including pulsation intensity, continuous phase flow rate, and dispersed phase flow rate, on the drop size distribution for the n-butyl acetate-water system. Figure 6a and b show that the phase flow rate has little to no effect on the drop size distribution. Increasing the dispersed phase flow rate slightly widens the drop size distribution due to a higher chance of coalescence. While, changes in the continuous phase flow rate have almost no impact on the drop size distribution. In contrast, Fig. 6c demonstrates that increasing pulsation intensity shifts the drop size distribution to the left. This occurs because higher pulsation intensity boosts collision energy and turbulence within the system. As a result, the frequency of eddy-drop collisions and drop breakup increases, leading to a higher probability of drop breakage^38^. Consequently, the distribution becomes narrower and more uniform, with an increased number of small drops.

**Fig. 7:**
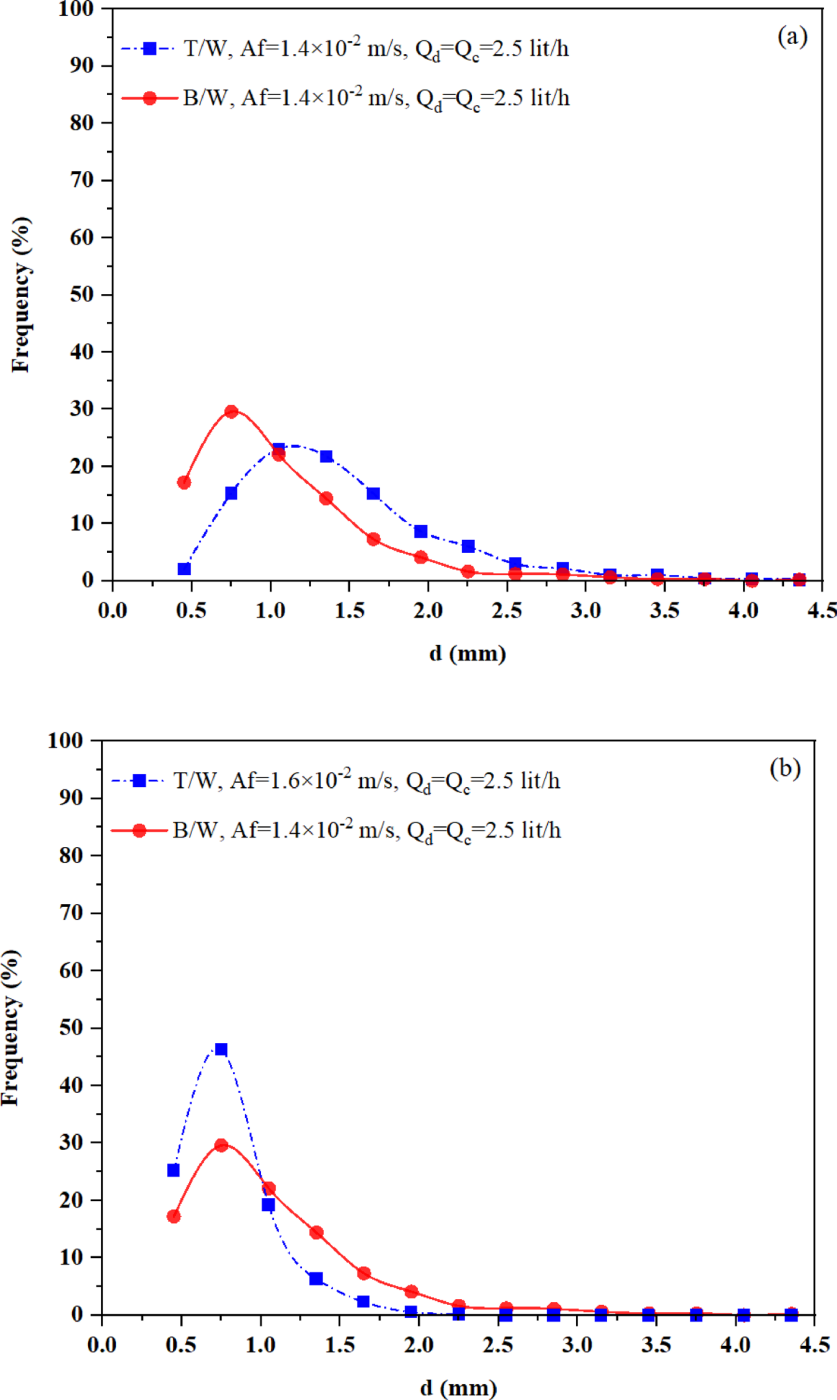
Effect of interfacial tension on drop size distribution for toluene-water (T/W) and n-butyl acetate-water (B/W) systems: (**a**) under the same operating conditions, and (**b**) under increasing pulsation intensity.

Figure 7 illustrates the effect of pulsation intensity on the drop size distribution for two different liquid–liquid systems. As shown in Fig. 7a, the system with lower interfacial tension, such as n-butyl acetate–water, exhibits a narrower distribution and a higher proportion of smaller droplets under identical operating conditions. Specifically, approximately 70% of the droplets in the n-butyl acetate-water system are smaller than 1 mm in diameter, compared to about 40% in the toluene-water system. This highlights the significant influence of interfacial tension at low pulsation intensities. However, Fig. 7b shows that as pulsation intensity increases, the role of interfacial tension diminishes. At higher intensities, mechanical forces become dominant, promoting the formation of smaller droplets with narrower distributions. Consequently, the drop size distributions of both systems converge, becoming nearly identical under sufficiently strong pulsation.

### Correlation of drop size distribution

In order to establish an appropriate correlation for drop size distribution, the log-normal distribution has been recognized as effective for describing drop sizes in extraction columns. Drop size distribution is identical in both the vertical and horizontal sections of the L-shaped packed column, therefore, a general correlation can be developed for both sections, as follows:

8 $$P_{\lg } (d) = (\frac{1}{{\sqrt {2\pi } d\alpha }})\exp \left[ { - \frac{{\left( {\ln (d) - \beta } \right)^{2} }}{{2\alpha^{2} }}} \right]$$

where $P_{lg} (d)$ is the frequency of drops in diameter of d, while $\alpha$ and $\beta$ are fitted parameters to be adjusted. AARE between experimental and predicted data was used to evaluate the accuracy of the fitted parameters. This metric was calculated using the following equation.

9 $$AARE = \frac{1}{n}\sum\limits_{i = 1}^{n} {\left( {\frac{{\left| {y_{\exp ,i} - y_{prd,i} } \right|}}{{y_{\exp ,i} }}} \right)}$$

The two fitted parameters were determined using the Minitab software, are presented in Table 8. As shown in this table, the fitted parameters $\alpha$ and $\beta$ have AARE values of approximately 7.5% and 12.6%, respectively, showing that these equations are recommended for practical application.

**Table 8:** Correlations and AARE values for fitted parameters in Eq. (8).

Equation	Conditions	AARE (%)
$\alpha = 5.03\left( {\frac{{Af^{4} \rho_{c} }}{\sigma g}} \right)^{0.199} \left( {\frac{\sigma }{{\rho_{d} \sqrt {Af^{3} Q_{d} } }}} \right)^{ - 0.296}$	No limitation	7.5
$\beta = 2 \times 10^{ - 6} \left( {\frac{{Af^{4} \rho_{c} }}{\sigma g}} \right)^{ - 1.479} \left( {\frac{\sigma }{{\rho_{d} \sqrt {Af^{3} Q_{d} } }}} \right)^{ - 0.377}$	$Af < 1.4 \times 10^{ - 2} {\text{m}}/{\text{s}}$ for B/W $Af < 1.5 \times 10^{ - 2} {\text{m}}/{\text{s}}$ for T/W	12.6
$\beta = - 1.55 \times 10^{ - 8} \left( {\frac{{\mu_{c} }}{{\rho_{d} \sqrt {Af_{ \, } Q_{d} } }}} \right)^{ - 3.794}$	$Af \ge 1.4 \times 10^{ - 2} {\text{m}}/{\text{s}}$ for B/W $Af \ge 1.5 \times 10^{ - 2} {\text{m}}/{\text{s}}$ for T/W	

### Prediction of drop size distribution: semi-empirical and ANN models vs experimental data

**Fig. 8:**
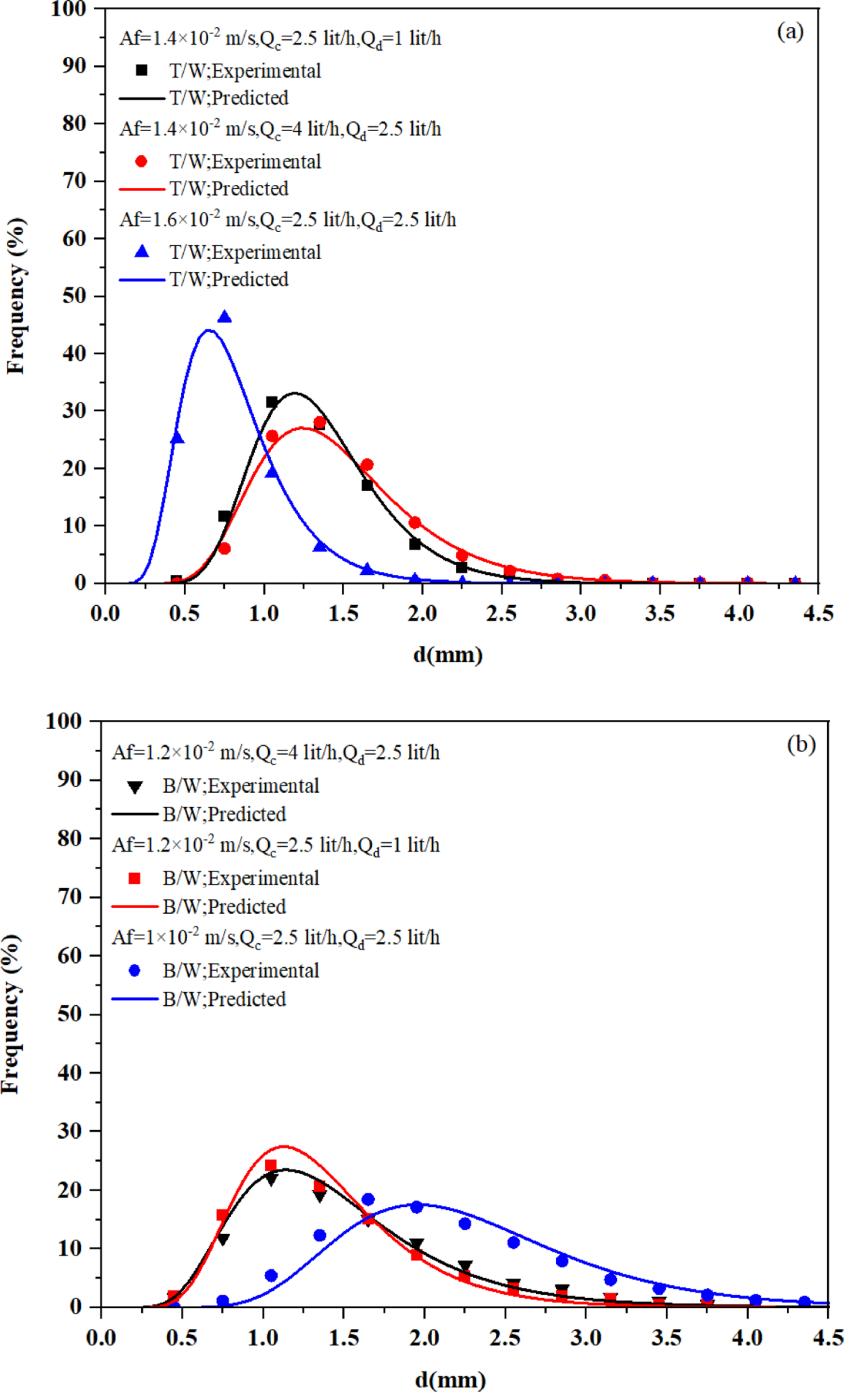
Comparison of experimental data with predictions from the derived correlation (Eq. (8)) under various operating conditions for (**a**) toluene-water (T/W) and (**b**) n-butyl acetate-water (B/W) systems.

Fig. 8a and b compare the correlation predictions of Eq. (8) with experimental data for toluene-water and n-butyl acetate-water systems under various operating conditions, respectively. Thes figures demonstrate that the correlation accurately predicts the experimental data, indicating its practical applicability.

**Fig. 9:**
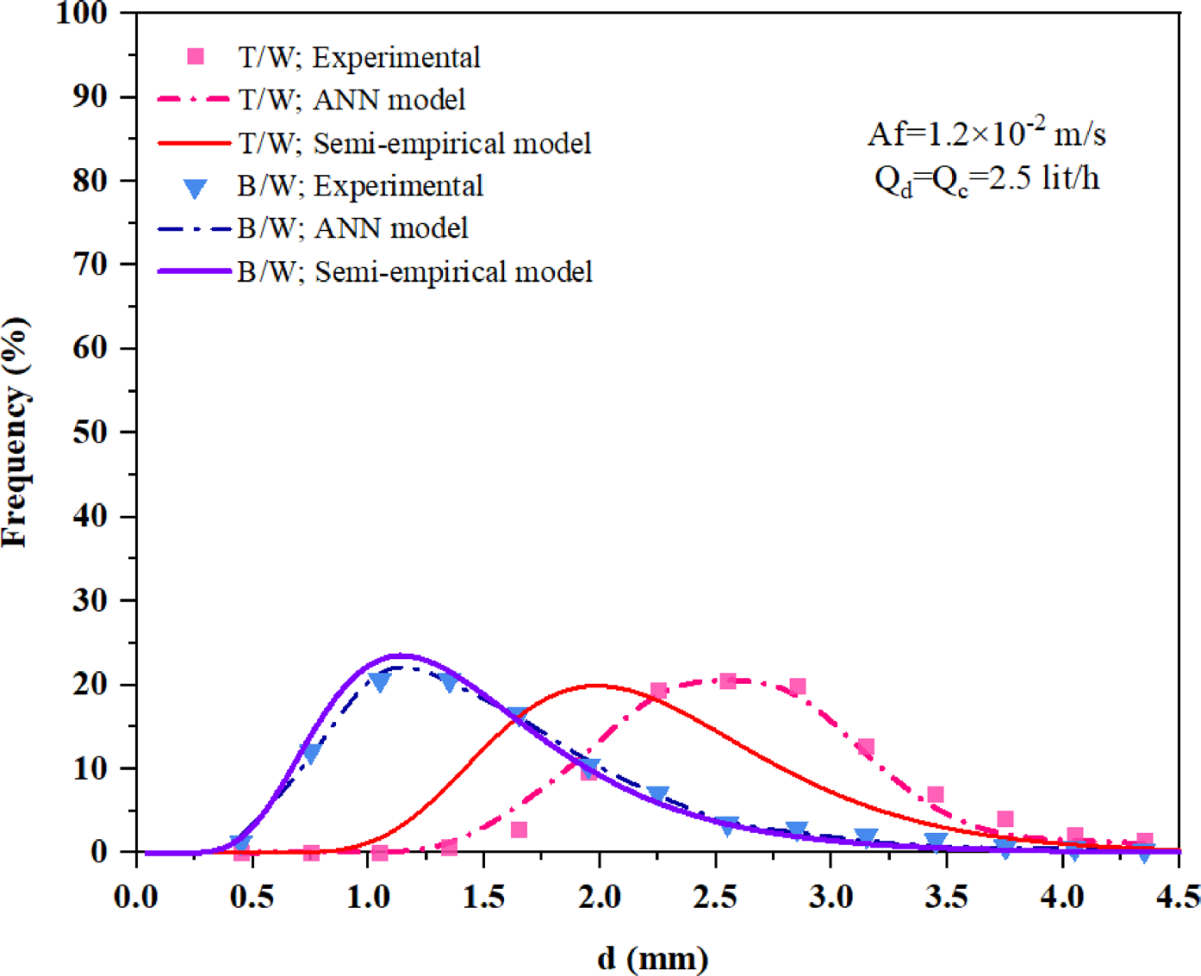
Comparison of experimental data with ANN and semi-empirical model predictions for two systems: n-butyl acetate-water (B/W) and toluene-water (T/W).

Figure 9 presents a comparison between the experimental data and the predictions from the ANN model for two systems: n-butyl acetate-water and toluene-water, under conditions of $Af=1.2\times {10}^{-2}\hspace{0.17em} m/s, Qc=Qd=2.5 lit/h$. The worst prediction of the semi-empirical model, observed for the T/W chemical system under the specified operating conditions, was selected for comparison with the ANN model. Despite this, the ANN model provided an excellent prediction of the drop size distribution behavior, demonstrating very high accuracy. The figure further shows that the ANN model offers a reasonable prediction of the drop size distribution for both systems, with better agreement between the ANN model and the experimental data compared to the semi-empirical model. This consistency highlights the ANN model’s capability to effectively capture the complex behaviors of liquid-liquid extraction systems.

### Mean drop size correlation

New semi-empirical correlations have been developed to estimate the mean drop size in both sections of the L-shaped packed column, considering the operating conditions and physical properties of the liquid systems. The following correlation, derived through dimensional analysis using Minitab software, is proposed for calculating the mean drop diameter in the column.

10 $$d_{32} = 0.036\left( {\frac{{Af^{4} \rho_{c} }}{\sigma g}} \right)^{ - 0.546} \left( {\frac{\sigma }{{\rho_{d} \sqrt {Af^{3} Q_{d} } }}} \right)^{ - 0.256}$$

**Fig. 10:**
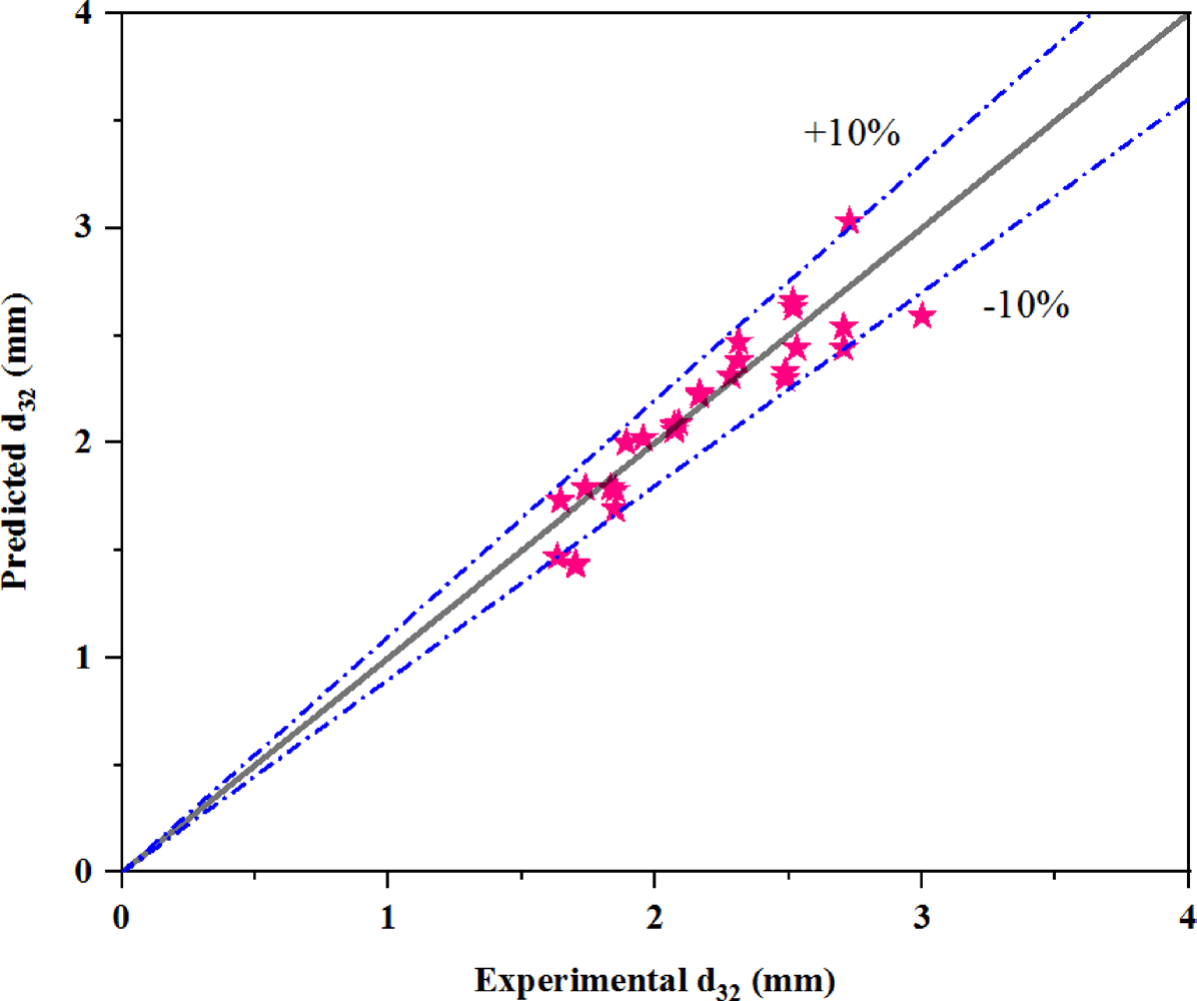
Comparison of mean drop size from experimental data with the semi-empirical correlation, as given by Eq. (10).

Figure 10 compares the mean drop size obtained from experimental data with the values predicted by Eq. (10). The accuracy of the proposed equation is validated by an AARE of 6.0%, demonstrating its reliability in predicting the mean drop size of the dispersed phase.

### Prediction of mean drop size: semi-empirical and ANN models vs experimental data

**Fig. 11:**
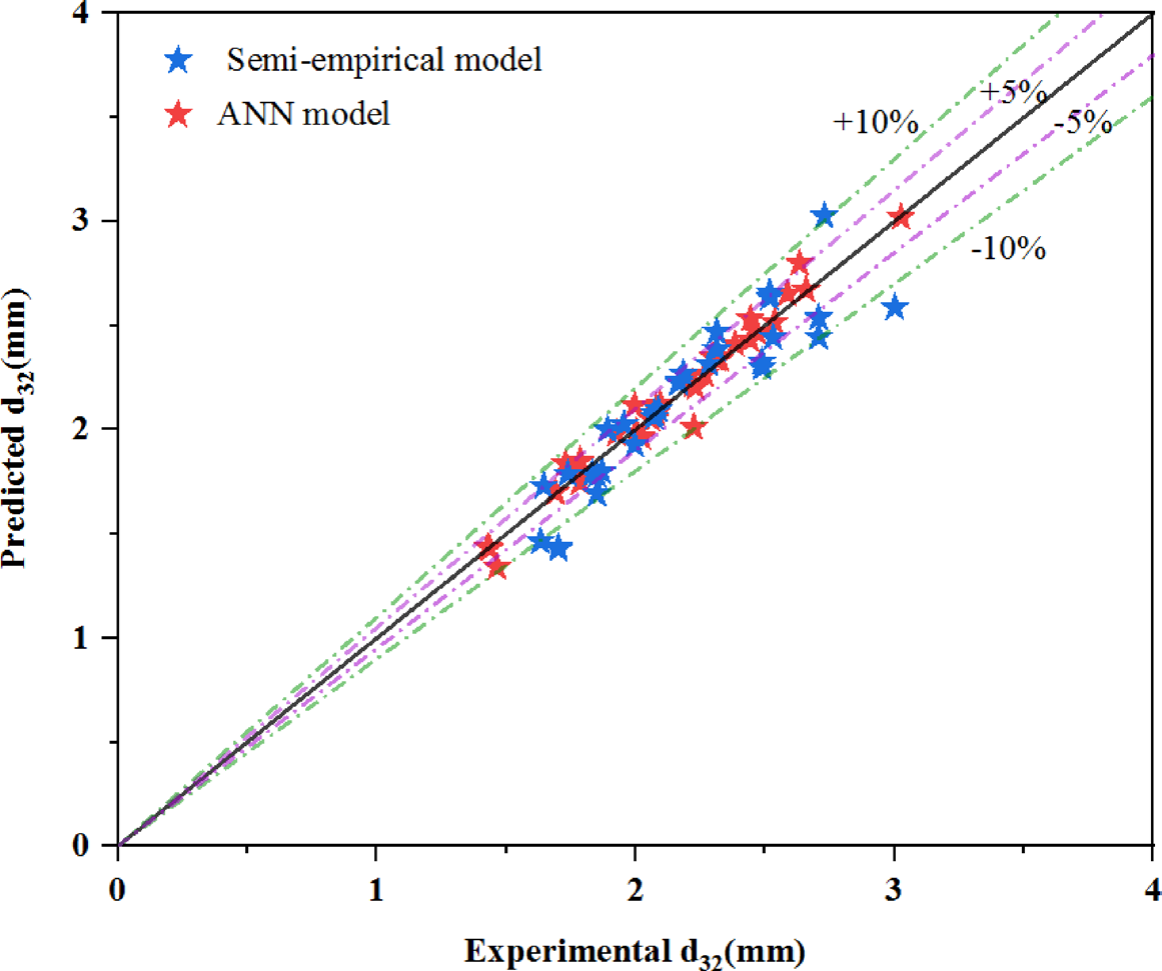
Comparison of ANN and semi-empirical models in predicting the mean drop size.

Figure 11 presents the semi-empirical and ANN models together. This figure demonstrates that the maximum error of the ANN model is half that of the semi-empirical model, with an AARE of 2%. Therefore, the ANN model provides better predictions across different operating ranges and physical properties.

Compared to existing ANN models developed for pulsed columns, the proposed ANN model exhibits better accuracy in predicting both mean drop size and its distribution. For instance, while Wang et al.^17^ reported an AARE of 9.6% and 18.5% for drop size prediction in PDDC and pulsed sieve plate column, the current model achieves an AARE as low as 2%, highlighting the advantage of integrating detailed experimental data and optimized network architecture.

### The effect of operation conditions and physical properties on the mean drop size

In this study, a sensitivity analysis was conducted to assess the effectiveness of each variable on the mean drop size using the developed ANN model. During the analysis, the performance was evaluated by ignoring each of the six input parameters in turn as shown in Table 9. This table shows that the operating parameters have a greater effect on the mean drop size compared to the property parameters. Pulsation intensity, among the operating parameters, has a significant impact on the mean drop size. Among the property parameters, interfacial tension has the greatest impact.

**Table 9:** Sensitivity analysis of each input variable on the mean drop size using the Levenberg–Marquardt algorithm with 10 neurons in the hidden layer for sensitivity analysis.

Parameter classification	Ignored parameter	MSE	R^2^
Operating Parameters	Pulsation intensity	0.1360	0.248
	Dispersed phase flow rate	0.0149	0.943
	Continuous phase flow rate	0.0062	0.977
Property parameters	Interfacial tension	0.0168	0.945
	Viscosity of dispersed phase	0.0113	0.956
	Density of dispersed phase	0.0113	0.956

**Fig. 12:**
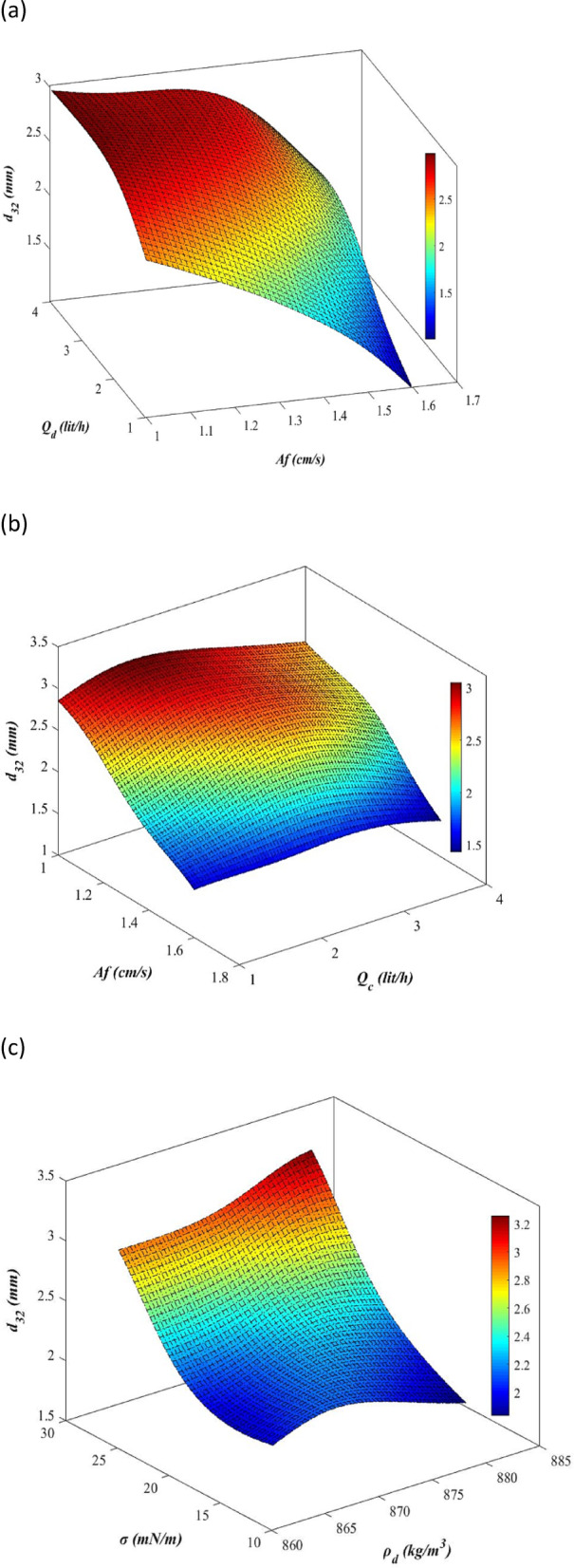

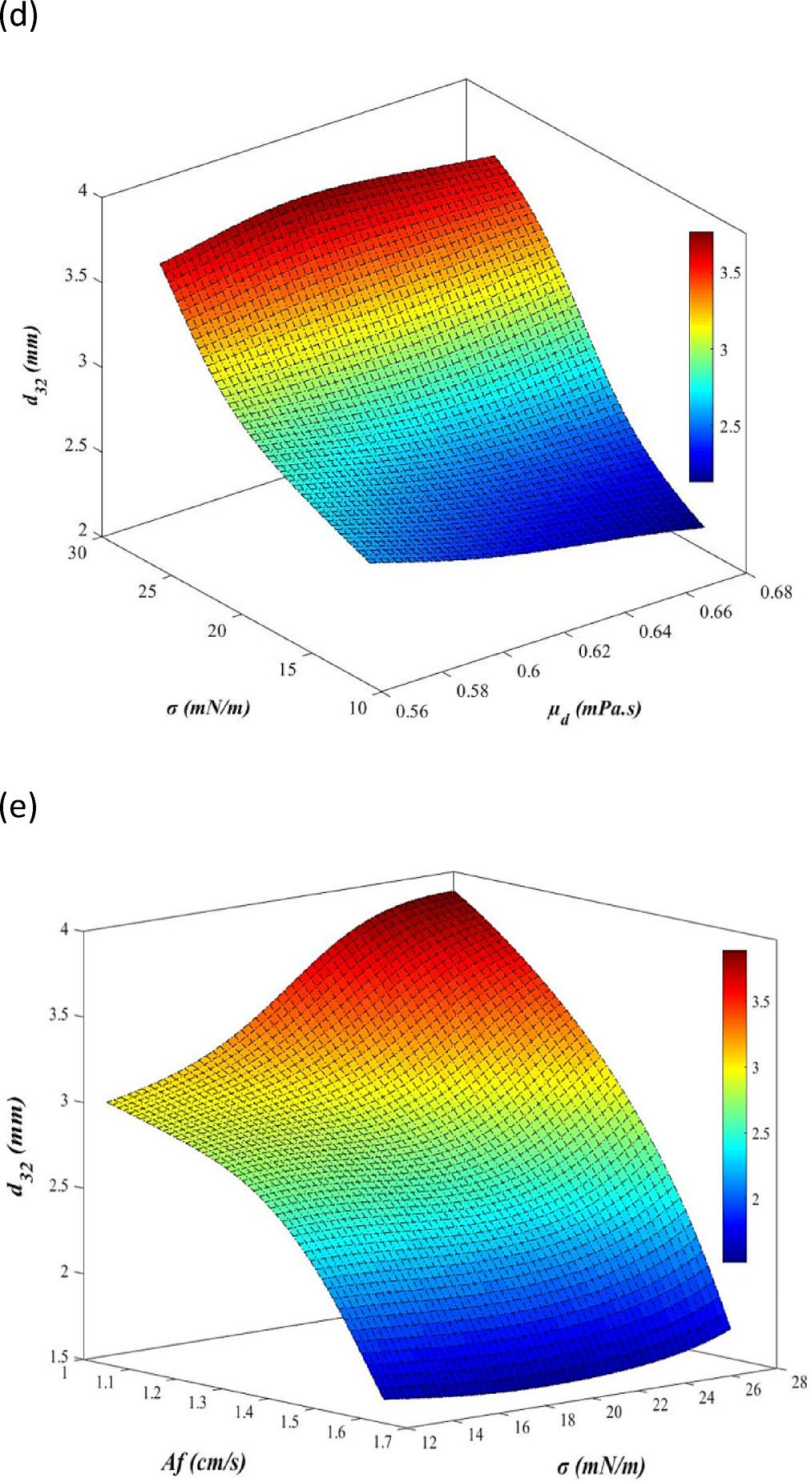
3D response surfaces of mean drop size as a function of: (**a**) pulsation intensity and dispersed phase flow rate, (**b**) pulsation intensity and continuous phase flow rate, (**c**) interfacial tension and dispersed phase density, (**d**) interfacial tension and dispersed phase viscosity, (**e**) pulsation intensity and interfacial tension.

For a clearer understanding of Table 9, Fig. 12a–e illustrate the analysis of operational and physical properties on the mean drop size in a 3D plots. Fig. 12a and b analyze the operational parameters, emphasizing pulsation intensity as the most influential factor compared to the flow rates of dispersed and continuous phases. Figure 12c and d investigate the physical properties, with interfacial tension having the greatest impact when compared to the viscosity and density of the dispersed phase. Figure 12e examines interfacial tension and pulsation intensity as the dominant physical property and operational parameter, respectively. As shown in this figure, the interaction effects of pulsation intensity and interfacial tension are analyzed. At higher pulsation intensities, the influence of interfacial tension becomes negligible. The results obtained from these plots confirm the data presented in Table 9.

## Conclusion

In this study, the drop size in an L-shaped pulsed packed extraction column was analyzed using an ANN model and newly developed semi-empirical correlations for two chemical systems: toluene-water and n-butyl acetate-water, in the absence of mass transfer. The ANN model was constructed using the Levenberg–Marquardt backpropagation algorithm, employing 10 neurons in the hidden layer for predicting the mean drop size and 9 neurons for the drop size distribution. The ANN model predicted the experimental data for drop size distribution and mean drop size, with maximum R^2^ values of 0.981 and 0.986, respectively. Furthermore, semi-empirical correlations for drop size distribution were developed based on a two-parameter log-normal probability function, which was found to effectively represent the experimental drop size distributions, with AARE values of approximately 7.5% and 12.6%. For the mean drop size, the ANN and semi-empirical models achieved AARE values of 2% and 6%, respectively. The effects of operating parameters and physical properties on drop size and its distribution were systematically examined. Pulsation intensity and interfacial tension were identified as the most influential factors. Higher interfacial tension increased drop size and widened the distribution, while higher pulsation intensity reduced drop size and narrowed the distribution. At elevated pulsation intensities, the differences in drop size distributions between the two chemical systems became negligible, leading to similar behavior. The findings of this study provided practical insights for the design and operation of L-shaped pulsed packed columns.

## Data Availability

All data associated with this study are presented within the manuscript. The raw datasets are available from the corresponding author upon reasonable request.

## List of symbols

*Af*: Pulsation intensity (m/s)
*A*: Pulsation amplitude (m)
*f*: Pulsation frequency (1/s)
d: Diameter of drop (m)
d_32_: Mean drop size (m)
n_i_: Number of droplets (–)
Q: Flow rate (lit/h)
P: Probability function (–)
g: Acceleration of gravity (m/s^2^)
u: Velocity (m/s)

## Greeks

*ρ*: Density (kg/m^3^)
*µ*: Viscosity (kg/m·s)
*σ*: Interfacial tension (N/m)

## Subscripts/Superscripts

*c*: Continuous phase
*d*: Dispersed phase
*lg*: Log normal distribution function
